# The Medical Use of Electricity

**Published:** 1867-02

**Authors:** G. M. Beard, A. D. Rockwell

**Affiliations:** New York; New York


### THE MEDICAL USE OF ELECTRICITY.

By G. M. BEARD, M.D. and A. D. ROCKWELL, M.D., New York.

The history of the medical employment of electricity has been marked by many and peculiar disappointments.

It was very natural to infer that an agent at once so mighty and mysterious in its phenomena, should have a great power for good or evil over the human constitution. Hence we find, that after the researches of Galvani had abundantly established the doctrine of the existence of animal electricity, very many enthusiastic observers set themselves to the task of demonstrating the medicinal virtues of this subtle fluid. Their experiments were attended with a measure of success. Aldini, in 1795; Hufeland, in 1798; Alibert, in 1817; and Dr. Mansford, in 1818, clearly establish the fact of the remedial powers of static electricity in certain forms of paralysis and epilepsy. The discovery of the induction current by the great Faraday, in 1831, gave a new impetus to the scientific investigations in this department. Matteucci, Du Bois Reymond, Golding Bird, Duchenne, Remak, and more recently, Brown-Séquard, Rosenthal, Meyer, Benedict, and Ziemssen, have all labored diligently in the field of physiological and medical electricity, and have brought many and valuable sheaves with them.

By these investigators, the remedial, as well as the physiological, effects of electrization have been repeatedly demonstrated. They have proved that, not only in paralysis and epilepsy, but also in cases of debility and impaired nerve energy, electricity is an agent of vast and wondrous power. And yet, outside of the ranks of these original explorers, there are comparatively few in the profession who have given the subject sufficient heed even to inform themselves as to the diseases for which galvanization or faradization are specially applicable. This apathy of the medical world with regard to the success of experiments that promise so much and so surely for the department of therapeutics, is to be accounted for by a variety of reasons. First of all, electricity, in the various methods in which it is employed, has not fulfilled the general expectation. It has been found to fail utterly in many cases where theoretically it should have achieved the most absolute success; and hence many, disappointed and perhaps disheartened, have illogically concluded that their expectations were not well grounded.

Again, in our country at least, the practical application of this agent has fallen into the hands of uneducated and unscrupulous practitioners, who know little of the human system, or of the science of medicine, and still less of the agent they employ. Emipirics and charlatans, versed in no art except that of robbing the unfortunate, have thus far had the field mostly to themselves, and have improved their advantage by filling their own pockets without adding an iota to the world's stock of experience. Whatever valuable truths they may have stumbled upon by their abundant observations are known only to themselves, and are regarded by them merely as tricks of trade, and in the very nature of things must die with them. Electricity appears to be travelling slowly in the footsteps of all our permanent specialities. Twenty years ago, the treatment of the diseases of women was almost exclusively in the hands of ignorant and unprincipled outsiders; gynecology is now one of the most honored and useful departments of science. It is but fifteen years since oculists were linked with contempt in the speech of all who desired to be regarded as authority in medical etiquette; to call oneself a specialist for the eye was a plea of guilty to the grossest ignorance and fraud.

Ten years ago, the diseased throats and ears of the country were at the mercy of a crowd of the most rapacious sharpers that ever amassed fortunes out of human suffering and credulity; ten years hence, laryngoscopists and aurists will stand on the same platform with oculists and gynecologists.

It is the duty, and it should be the delight, of scientific men, to wrest the medical employment of electricity from the hands of these selfish harpies, and accord to it that honor to which its merits justly entitle it. At the present time medical practitioners of all grades are not unwilling to recommend electricity in certain cases of paralysis, that will neither yield to internal medication nor get well in spite of it; but they usually allow their patients to use some kind of apparatus at home, or else content themselves with two or three imperfect applications under their own eyes. The results in such cases are almost always unsatisfactory. It could not, indeed, be otherwise. In this way, more than in any other, electricity has been wounded in the homes of its friends. To one case that is cured or relieved by such slipshod procedures, ten are either made worse, or else so little benefitted that they cast their batteries aside, and ever afterwards declare that electricity is a humbug. The truth is, that there is no more sense or reason in allowing patients to make their own applications of the electric current, than there would be in intrusting them with the responsibility of cutting their own veins, or operating on their own ears and eyes. In three important particulars we are apt to mistake the employment of electricity. 1. By neglecting to make just discrimination in regard to the types and phases of disease that are found to yield most readily and surely to this method of treatment. 2. By intrusting the details of the applications to the patients themselves, or some of their non-professional attendants. 3. By not making the applications with sufficient thoroughness and persistence.

Our attention having been called to this subject for some time past, we have found that the range of diseases amenable to this form of treatment is much wider than we had ever supposed. We have found that faradization, or the use of the secondary current, is especially indicated in cases of indigestion in all its myriad shapes; in nervous derangements, when they take the form of chorea, epilepsy, neuralgia, or hypochondriasis; and in general debility and anæmia, dependent on any cause except pulmonary tuberculosis. In our experiments thus far we have made use of Smee's battery, as manufactured by Kidder, of this city, and we have employed in most cases only the secondary current. It is very far from being a fixed fact of medical science that the primary current is capable of succeeding when the secondary fails; but, if it be used at all, it is necessary to obtain the combined strength of a number of elements. The primary current of the battery we use is too weak to be of any special service in ordinary cases, and we have never secured from it any results that could not have been obtained just as surely, and far more rapidly, by faradization. Stöhrer's large battery, consisting of twenty-four or thirty-two elements, is a most excellent instrument, and is much employed by Ziemssen, Rosenthal, and other German investigators.

Three different methods of applying electricity have been recommended by Duchenne; by solid metallic electrodes, metallic brushes, and the hand.

Of these methods we must prefer the latter.

No instrument that human skill shall devise can ever equal the hand in flexibility and power of adaption. If the feet of the patient be placed on a sheet of copper to which the negative pole is attached, the operator, holding the positive pole in one hand, can with the other readily manipulate the parts desired to be affected, and by increasing or diminishing his grasp of the sponge, can modify the strength of the application without disturbing his apparatus.

Used in this way, the current must pass through the body of the operator. The first essays of those who may employ electricity through their own persons, must always be unsatisfactory. They will find that they can bear only a very feeble current, or at least one not strong enough to effect any but the weakest patients, or the most sensitive localities. But practice is everything here, as in the use of all other appliances of medicine. According to our observation and experience, the system appears to become accustomed to the powerful electric stream, just as it becomes accustomed to the use of tobacco, alcohol, opium, hasheesh, or coca; with this difference, that its effects are, if anything, positively beneficial.

Dr. Garratt, of Boston, (whose abundant opportunities for observation and varied practical experience in the medical employment of electricity, entitle his views to more consideration than those who have attempted to wade through the verbose and mystic rhetoric of his recent work will be willing to accord to him), advises "that the operator use the same hand that holds the electrode, so as to prevent the passage of so high an induction current through his own person, which is thus to himself highly injurious and unsafe to be long continued or often repeated."

It would be very natural to infer that an agent so potent in the cure of disease must be prejudicial to health when used in large excess, for such is found to be the case with nearly all the prominent articles of the materia medica.

It is on this probability that Dr. Garratt bases his words of warning; but the facts are against him. Obstinate experience will not wheel into line at the command of any scientific theory, however consistent or plausible.

We have now been employing faradization through our own persons for some time, and the effects have thus far been either negative or beneficial.

We have both enjoyed our average health since we began to use the agent, and both have observed a marked development of the strength and size of the muscles of the arm. Wm. Miller, of this city, a man of no special medical education, but of the utmost reliability, and thoroughly experienced in the practical application of the faradaic current, informs us that for the past thirty-five years he has allowed the stream to pass through his own body on an average of about five hours each day. By mathematicial computation, then, it appears that a powerful induced current of electricity has been passing through him for about seven years of his life. Up to the present time, his general health has been excellent, has, indeed, improved under the mighty stimulus, and he has suffered from no disease that can even be remotely ascribed to electricity. It is safe to say that no parallel instance can be found in either hemisphere.

Our experience thus far seems to have taught us three important facts:—

1. Faradization is a *tonic* of vast and varied powers, and it is chiefly through its tonic effects that it so rapidly and so surely benefits so many chronic asthenic diseases. It almost uniformly relieves chorea, dyspepsia, jaundice, constipation, neuralgia, and chronic rheumatism; also, anæmia, when dependent on functional nervous derangement; and when faithfully and persistenly employed, it not unfrequently works a permanent cure. Whether these tonic effects of faradization are the result of its mechanical action, or of some subtile nerve power that it mysteriously imparts to the system, or of both combined, we are of course unable to say. Nor is the question a vital one, however interesting it may be to the inquiring spirit of science.

The operators of the Atlantic cable inform us that enough electricity can be generated in a vessel no larger than a gun cap, to send a message from continent to continent, and they are ready to confess that they know as little of the nature of this agent as they did when Morse first planned the line to Washington.

Precisely the same principle holds good in the medical employment of electricity. Our ignorance of the rationale of its workings is no bar to our progress in the knowledge of its effects.

2. To gain satisfactory results from faradization in long standing cases of debility from whatever cause, the applications must be properly made and thoroughly persisted in. After the pendulum has been swinging for years in one direction, it does not make the return beat in an instant.

In employing clectricity, just as in the use of medicaments generally, the time required to complete a cure must bear some proportion to the duration of the malady.

3. For the successful employment of electricity in the various diseases for which it is applicable, there is need of much more skill, patience, and experience than is commonly supposed. It cannot be too often repeated, line upon line, and precept upon precept, that no specialty in science, however restricted may be its scope, can be thoroughly mastered without a good measure of skill, energy, and patience. And in regard to this very humble department of electricity, in the selection and care of the apparatus, in the wise discrimination between the cases which are and those which are not amenable to this method of treatment, in the acquiring of the requisite facility and effectiveness of application—in the entire mastering of the whole subject, there is a wide range for the exercise of scientific genius and diligence, and as imperious a necessity for large and varied experience, as in any other department of therapeutics.—*Medical Record, N. Y.*

